# Advances in Localized Prostate Cancer Management

**DOI:** 10.1155/2018/2807813

**Published:** 2018-02-04

**Authors:** David B. Samadi, Michael A. Feuerstein, Seyed Behzad Jazayeri, Steven A. Kaplan, Yasser Farahat

**Affiliations:** ^1^Department of Urology, Lenox Hill Hospital, Zucker School of Medicine at Hofstra/Northwell, New York, NY, USA; ^2^Department of Urology, Icahn School of Medicine, New York, NY, USA; ^3^Department of Urology, Faculty of Medicine, Tanta University, Tanta, Egypt

Advances in technology, including new imaging modalities, genomic tests, and biomarkers, have introduced practice-changing opportunities for the treatment of localized prostate cancer. These advances will allow us to better determine which patients are appropriate candidates for active surveillance and when to offer patients curative treatment. With the rising use of MRI-guided biopsy, further questions about when, and on whom, to use MRI-guided biopsy have been raised. In a quest to highlight best practices for achieving best cancer detection rates for patients with previous negative biopsies, C.-H. Chang et al. compared the prostate cancer detection rates of targeted biopsy and saturation biopsy in patients with previous negative biopsy and the accuracy of these biopsies retrospectively stratified by different serum prostate-specific antigen (PSA) levels. Of the 185 patients enrolled in the study, they found that combining target biopsy (TB) and saturation biopsy (SB) achieved the best cancer detection rate. Furthermore, the accuracy of TB was better than that of SB in patients with serum PSA > 10 ng/mL. More work is needed to evaluate different biopsy strategies in patients with varying PSA levels. Using a recently released robot guided, software based transperineal approach, M. Kroenig et al. compared prostate cancer detection rates between MRI-TRUS fusion targeted and systematic biopsies. 52 patients with elevated PSA levels, clinical suspicion for prostate cancer, and prior negative 12-core transrectal ultrasound guided biopsy received MRI/TRUST fusion biopsy at the University of Freiburg Medical Centre. This group was unable to exhibit an advantage in the overall detection rate of clinically significant prostate cancer using the MRI/TRUS fusion biopsy. This study highlights the potential to improve the radiological PI-RADSv2 classification scheme in order to reduce sensitivity issues.

Is it safe to offer young patients active surveillance? What are the long-term side-effects of treatment that young men might face? In this special issue, D. Milonas et al. report long-term outcomes of young men treated with radical prostatectomy. They compared long-term outcomes of 277 men aged ≤55 years after radical prostatectomy (RP) with an older cohort. Pathological tumor characteristics, biochemical recurrence rates (BCR), and disease progression rates were compared between the two groups. This group found that the younger cohort had superior outcomes especially when their Gleason score, lymph-nodes, and surgical margins status were lower, and the two cohorts had similar BCR. This study highlights the excellent results that RP has for younger men (≤55 years) with localized prostate cancer.

Treatment advances will also aid in a personalized approach to treatment, giving patients more information about their cancer and expected cancer and side-effects of any given treatment. E. Reamer et al. discuss the role of social support and personality differences in men when it comes to prostate cancer treatment selection. In their report, E. Reamer et al. analyzed social influences on the treatment decision-making process of 559 men ≤ 75 years old newly diagnosed with localized prostate cancer. A population-based sample was surveyed cross-sectionally from Detroit, Michigan, and cases were identified by the Metropolitan Detroit Cancer Surveillance System (MDCSS). They evaluated treatment choice, reason for the choice, decision-making difficulty, satisfaction, and regret. They found that, in addition to seeing a specialist, consulting friends increased men's likelihood of choosing curative treatment and that consulting family or friends increased decision-making difficulty. With a racially diverse cohort, this study highlights the importance of social networks during a patient's treatment decision-making process, and it has implications for how physicians should develop realistic expectations of treatments across communities.

And in an effort to increase awareness about the developments in definitive radiotherapy and compare its outcomes to radical prostatectomy, B. G. L. Vanneste et al. wrote a narrative review on a number of publications regarding definitive treatments for prostate cancer. The current literature did not reveal significant difference between conventional, definitive treatment modalities in cure rates, but in toxicity patterns. The paper focuses its conclusions on patient-specific treatment and recommending different treatment types based on their own advantages and side-effects in correspondence to the specific needs and concerns of individual patients.

